# Endocrine sequelae of hematopoietic stem cell transplantation: Effects on mineral homeostasis and bone metabolism

**DOI:** 10.3389/fendo.2022.1085315

**Published:** 2023-01-12

**Authors:** Francesca Miglietta, Luca Iamartino, Gaia Palmini, Francesca Giusti, Francesca Marini, Teresa Iantomasi, Maria Luisa Brandi

**Affiliations:** ^1^ Department of Experimental Clinical and Biomedical Sciences “Mario Serio”, University of Florence, Florence, Italy; ^2^ Fondazione FIRMO Onlus (Italian Foundation for the Research on Bone Diseases), Florence, Italy

**Keywords:** hematopoietic stem cell transplantation, mineral homeostasis, bone metabolism, osteoporosis, bone resorption

## Abstract

Hematopoietic stem cell transplantation (HSCT) is an established therapeutic strategy for the treatment of malignant (leukemia and lymphoma) and non-malignant (thalassemia, anemia, and immunodeficiency) hematopoietic diseases. Thanks to the improvement in patient care and the development of more tolerable conditioning treatments, which has extended the applicability of therapy to the elderly, a growing number of patients have successfully benefited from HSCT therapy and, more importantly, HSCT transplant-related mortality has consistently reduced in recent years. However, concomitantly to long term patient survival, a growing incidence of late HSCT-related sequelae has been reported, being variably associated with negative effects on quality of life of patients and having a non-negligible impact on healthcare systems. The most predominantly observed HSCT-caused complications are chronic alterations of the endocrine system and metabolism, which endanger post-operative quality of life and increase morbidity and mortality of transplanted patients. Here, we specifically review the current knowledge on HSCT-derived side-effects on the perturbation of mineral metabolism; in particular, the homeostasis of calcium, focusing on current reports regarding osteoporosis and recurrent renal dysfunctions that have been observed in a percentage of HSC-transplanted patients. Possible secondary implications of conditioning treatments for HSCT on the physiology of the parathyroid glands and calcium homeostasis, alone or in association with HSCT-caused renal and bone defects, are critically discussed as well.

## 1 Introduction

Hematopoietic stem cell transplantation (HSCT) is today a well-recognized treatment for many neoplastic [i.e. leukemias, lymphomas (1–5)] and non-neoplastic [i.e. thalassemia, anemia (6, 7)] hematological diseases (**Table 1**), performed by an infusion of hematopoietic stem cells (HSCs) that can be collected from bone marrow, cord blood, and peripheral blood (8–10).

**Table 1:** Conditions for which patients may undergo HSCT.

Malignant/neoplastic diseases	
Leukemias	Acute myeloid leukemia (AML)
	Acute lymphocytic leukemia (ALL)
	Chronic myeloid leukemia (CML)
	Myelodysplastic/Myeloproliferative neoplasms (MDS/MPS)
	Chronic lymphocytic Leukemia (CLL)
	Other leukemias
Lymphoproliferative diseases (LPD)	Plasma cell disorders
	Hodgkin/Non-Hodgkin lymphoma HD/NHL
Solid tumors	Kidney
	Pancreas
	Thymus
	Breast
	Neuroendocrine tumors
	Other solid tumors of unknown primary origin
Non-malignant disorders	
Bone marrow failures	
Hemoglobinopathies	
Immunodeficiencies	
Inherited diseases of metabolism	
Autoimmune disorders	
Thalassemia	
Anemia	
Other non-malignant disorders	

Two types of HSCT can be performed, autologous or allogeneic transplantation, both leading to remission of bone marrow aplasia (11–13). In autologous HSCT, HSCs from the same recipient are collected and cryopreserved. After a conditioning treatment, the patient receives an infusion of his/her own HSCs (8). Allogeneic transplantation, instead, consists of the infusion of HSCs isolated from a healthy donor, who can, preferentially, be a half-HLA-matched biological parent or biological children, or, in absence of these first-degree relatives, a partially-HLA-matched family member or unrelated donor (14). Graft versus host disease (GVHD) represents a severe adverse reaction of the allogeneic transplantation, caused by the targeting of the host non-hematopoietic cells by the T lymphocytes of the donor. To minimize the risk of GVHD a stringent HLA matching between recipient and donor is strictly required. On the other hand, allogeneic transplanted HSCs have been showed to exert immunological effects against the malignant hematopoietic cells of the recipient, the so-called graft versus tumor GVT) reaction, due to donor’s polyclonal T-cell response targeting polymorphic antigens expressed on the recipient’s hematopoietic tissue. This characteristic made the allogeneic HSCT applicable also to elderly and clinically infirm patients, who were previously not candidates for the autologous transplantation because of the requirement of a high-dose of pre-transplantation conditioning treatment.

From 1957 to 2016 a total of 1,298,897 HSCTs were reported, of them 57.1% being autologous procedures (15). Approximately 90,000 first HSCTs are performed yearly worldwide and this number is estimated to increase by 10-20% annually. Autologous transplantations represent about 53% and allogeneic about 47%, of which about 53.6% are from related donors (15). In 2016, the World Health Organization reported a global increase of 6.2% in autologous HSCT and 7.0% in allogeneic HSCT (15). Today, HSCT is successfully performed in almost all age patients. A recent report by the European Society for Blood and Marrow Transplantation (EBMT) (16), resuming activity and trends on HSCT in 42 European and 11 collaborating countries, reported a total of 48,512 HSCTs (59% autologous and 41 allogeneic) of which 5,189 (10.7%) were performed in pediatric patients less than 18 years (76.9% allogeneic and 23.1% autologous). Main indications for allogeneic HSCT were myeloid malignancies, while for autologous transplantation were lymphoid malignancies.

Before receiving the intravenous infusion of HSCs, the recipient must undergo a conditioning treatment, consisting of chemotherapy and/or radiation therapy, in the 3-5 weeks prior to transplantation, leading to the ablation of the patient’s ailing bone marrow and the induction of sufficient immunosuppression to allow transplantation of HSCs, preventing the risk of rejection (8). The conditioning regimen can be myeloablative (full-dose) or non-myeloablative (reduced-intensity) (9).

The intensity and choice of conditioning regimen varies depending on the diagnosis and remission status of the disease, the presence or absence of comorbidities, the patient’s age and donor availability. The myeloablative regimen consist of therapy with alkylating agents, with or without total body irradiation (TBI), and it is designed to completely ablate bone marrow hemopoiesis, not allowing autologous hematological recovery. In contrast, non-ablative regimens, although causing cytopenia, do not require stem cell support (17).

Due to its immunosuppressive property, TBI is widely used as a conditioning regimen for patients with hematological malignancies undergoing HSCT. Generally, the regimen consists of a combination of 12 to 16 Gy TBI (usually hyperfractionated in order to decrease the risk of side effects, such as interstitial pneumonitis) (18, 19) with chemotherapeutic agents, such as cyclophosphamide (20–23), cytarabine (AraC) (24), etoposide (25), melphalan (26) and busulfan (27).

To avoid the short-term/long-term toxicity effects caused by high-dose TBI (especially in patients already treated with radiotherapy), alternative conditioning regimens can be used, having similar immunosuppressive efficacy (28, 29) based on the administration of chemotherapeutic agents, instead of TBI, such as alkylating agents. Busulfan is an alkylating agent with a strong toxic effect on non-dividing bone marrow cells, but it cannot be administered as the only treatment due to its low effect against mature lymphocytes (21). The regimen that is generally used on patients undergoing HSCT (both autologous and allogeneic) consists of high-dose busulfan (16 mg/kg total dose) and cyclophosphamide (120 mg/kg) (30). More recently, to further reduce regimen-dependent toxicity, cyclophosphamide has been replaced by treatment with fludarabine (a nucleoside analogue with immunosuppressive activity that synergizes with alkylating agents by inhibiting DNA repair (31, 32).

Reduced-intensity allogeneic HSCT results in a variable degree of initial donor-host mixed chimerism (21) that may vary depending on the intensity of the actual conditioning regimen, donor/host HLA disparity and transplant composition (33). The efficacy of GVT effects may also be influenced by tumor burden and proliferation rate of the neoplasm (33). To date, there are various types of non-ablative approaches based on a low-intensity TBI in combination with fluradabine or cloforabine followed by treatment with mycophenolate mofetil and a calcineurin inhibitor ± sirolimus, such as in the cohort studies published by Cooper et al. (34); or such as those developed at the MD Anderson Cancer Center (35), where a treatment approach based on a non-myeloablative regimen containing fludarabine (90 mg/m^2^) and cyclophosphamide (2,250 mg/m^2^) in combination with rituximab is suggested in order to prevent transplant rejection and increase host T-cell immunosuppression.

After HSCT, patients must follow immunosuppressive therapy consisting in corticosteroids and calcineurin inhibitors to prevent the occurrence of GVHD.

Currently, over 90,000 HSCTs are yearly performed worldwide, and, thanks to the development of more tolerable conditioning treatments, long-term survival of patients has been increasing along the year (36), with an estimated survival rate beyond 10 years after transplantation of approximately 80% (37). The mortality rate is due to various complications associated with the transplant itself, including acute and chronic GVHD, infections, and end-organ dysfunction (38). Moreover, as the post-transplant long-term survival has increased, there has been an increasing incidence of sequelae, impacting on mortality rate and morbidity, and worsening patient quality of life. One of the HSCT-derived main complications is the occurrence of endocrine alterations that impair the health of transplanted patients, with variable degrees of severity (39). Conditioning chemotherapy and radiotherapy, and post-operative immunosuppressive treatments are the principal causes of hormone dysfunctions, and subsequent development of endocrine syndromes, after transplantation (40, 41). As a consequence of these sequelae, transplanted patients require lifelong care for endocrine disease management.

Here, we review adverse events and sequelae caused by HSCT, and associated conditioning and post-transplantation immunosuppressive therapies, specifically altering local and systemic endocrine regulation of bone and mineral homeostasis.

## 2 HSCT-derived endocrine sequelae pathophysiology

### 2.1 Altered mineral homeostasis and renal dysfunction

Myeloablative and non-myeloablative conditioning media and cyclosporine treatment can create significant damages to patients by affecting electrolyte balance, through the alteration of the balance between intestinal absorption and renal excretion (42–44). Serum levels of calcium, magnesium, potassium, and phosphorus, in fact, tend to drop suddenly, thus creating a severe acid/base imbalance (44). Philibert et al. (43) observed that in 48 patients receiving HSCT from peripheral blood, 40% had serum potassium values less than 3.5 mmol/l, 33% had magnesium values less than 0.7 mmol/l, 43% had calcium values less than 2.14 mmol/l, and 35% had phosphorus values less than 0.6 mmol/l, immediately after HSCT. In total, 91% of patients had hypophosphatemia, while 49% had hypocalcemia after the transplantation. Crook et al. (44) reported severe hypophosphatemia in 77% of patients during the period of the conditioning regimen, or in the following week.

The hematopoiesis of transplanted cells may contribute to altering the phosphorus balance in patients undergoing HSCT, causing hypophosphatemia (45, 46); a diagnosis of severe hypophosphatemia after transplantation is directly associated with the proliferation of transplanted HSCs (44–48). Extensive phosphate consumption seen in HSCT patients is due to the need of transplanted HSCs of an energy source for the *ex-novo* proliferation of the hematopoietic cell compartment (44–48). Raanani et al. (45) observed a correlation between the release of the cytokines IL-6 and IL-8 and the severity of hypophosphatemia, defining the increase in these interleukins as a predictive index directly proportional to the severity of hypophosphatemia.

Hypomagnesemia observed in patients with HSCT may be one of the causes of altered electrolyte balance. Very low levels of magnesium cause a decrease in cAMP activity, which leads to prolonged opening of potassium channels, causing a decrease in serum levels of potassium itself (43, 49).

In addition, several studies (43, 44, 48, 50–53) have shown that intracellular magnesium depletion can compromise the functionality of parathyroid glands to secrete parathyroid hormone (PTH), through modulation of cAMP activity. Decreased PTH secretion, caused by hypomagnesemia, leads to hypocalcemia, and is associated with decreased active vitamin D (43, 49, 51, 52). Agus et al. observed that PTH-induced calcium release is severely impaired when the magnesium concentration is less than 0.8 mEq/l, and in severe hypomagnesemia there is resistance to PTH by target organs (49). It should be noted, however, that with magnesium replacement therapy, PTH serum levels are restored (42, 43, 52, 53).

**Figure 1:**
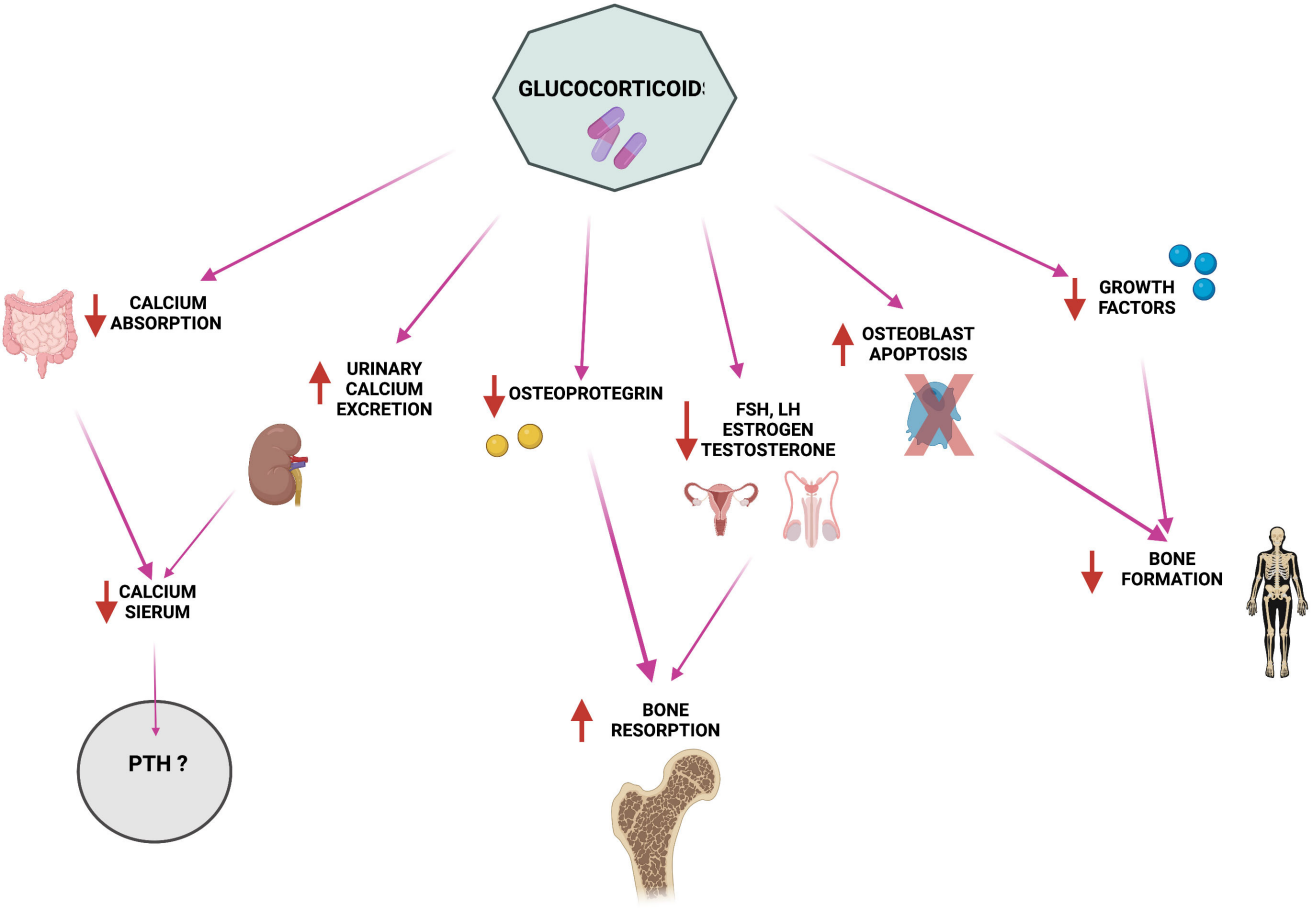
Pathophysiology of glucocorticoid-induced alterations in calcium homeostasis and bone metabolism. Created by BioRender.com.

Glucocorticoid therapy is the standard therapy against GVHD in allogeneic HSCT; these molecules alter PTH metabolism and calcium homeostasis. Among major side effects of glucocorticoids there are the suppression of osteocalcin (OCN) and alkaline phosphatase (ALP) expression, the induction of osteoclastogenesis and of bone resorption, and the impairment of osteoblastogenesis and osteoblastic activity, all causing severe bone loss and, consequently, osteoporosis (54). At the same time, glucocorticoids cause decreased intestinal calcium absorption and increased calciuria (**Figure 1**), causing a severe decrease in serum calcium levels (55, 56) and, thus, inducing a strong stimulation of PTH production and leading to the establishment of secondary hyperparathyroidism (55, 57, 58). However, studies related to this topic show mixed results. There are studies in which no correlation has been found between glucocorticoids (even at high concentrations) and PTH increase (59–64), whereas some reports have observed that there is a dose-dependent PTH increase during treatment with glucocorticoids. A study using Northern blot analysis was conducted to determine whether PTH release in humans was directly stimulated by glucocorticoids (65). Dexamethasone was found to increase the amount of preproPTH mRNA in cells by directly acting at gene level in parathyroid cells, increasing PTH synthesis through stimulation of PTH gene transcription (65).

The reduction in intestinal calcium absorption and the sharp increase in PTH serum levels (66) could be also synergistically caused by the interference of post-transplant long-term glucocorticoid therapy with vitamin D metabolism, creating a non-competitive effect on the vitamin itself (67, 68). Indeed, after transplantation, vitamin D levels drop further in 90% of patients, a severe decrease that was mainly related to the intake of glucocorticoids, rather than myeloablative conditioning treatment. Physiologically, vitamin D stimulates intestinal absorption of calcium and consequently promotes a decrease in PTH production and release by the parathyroid glands. In HSCT patients, concomitant lack of exposure to the sun, due to their disease status, and long-term therapy with immunosuppressive glucocorticoids induce an overall vitamin D deficiency that affects the correct calcium and phosphate homeostasis and favors the occurrence of osteopenia and osteoporosis (66).

Calcium and mineral metabolism may be further altered due to renal failure as a common complication following HSCT, causing profound effects on morbidity and long-term survival of patients. Chronic kidney disease (CKD), a late-stage gradual loss of kidney function, is commonly diagnosed after HSCT. In observational studies, CKD has shown a variable incidence in HSCT patients, varying between 18-66%, due to the different parameters used for CKD definition and the differences in the duration of follow-ups (69–71). In general, renal dysfunctions are multifactorial complications that can be caused by pre-transplant underlying diseases and comorbidities, conditioning regimens, transplant complications and prophylaxis treatments (72).

Similarly, acute kidney injury (AKI) is a severe renal complication characterized by defects in glomerular filtration. It is diagnosed in HSCT recipients with variable incidence depending on the type of HSCT [from 20% to 73% (73)], and on the administration of myeloablative and non-myeloablative conditioning treatments (70, 74–77).

Pre-existing renal pathologies, such as glomerulonephritis, develop from hematological malignancies for which HSCT intervention is planned, therefore, the underlying hematological diseases already provoke renal lesions before HSCT, increasing in this way the risk of severe renal failure in the long term HSCT survival (78). Moreover, comorbidities such as diabetes, hypertension, and arteriosclerosis have been shown to further contribute to renal failure after transplant (72).

Pre-operative conditioning regimes may damage the kidneys. Several chemotherapeutics have been reported to induce renal failure, such as pre-renal azotemia and acute tubular necrosis, due to their nephrotoxicity, so a reduction of their dosage in HSCT recipients has been recommended (72, 79, 80).

Several studies reported in HSCT patients undergone total body irradiation the occurrence of renal defects, diagnosed several years after the radiation exposure, due to the late effects of radiotherapy on kidney physiology (69, 72, 81–83). Typical histopathological features of radiation-induced nephropathy are vascular, glomerular, and tubulo-interstitial damages. Radiations cause DNA double-strand breaks resulting in apoptosis or necrosis of renal endothelial, tubular and glomerular cells (84).

Moreover, both chemotherapeutics and radiation therapy have been associated with hepatic sinusoidal obstruction syndrome, due their cytotoxic effects on hepatic sinusoidal endothelial cells. This HSCT complication has been seen to induce systemic detrimental effects, including AKI (85).

It has been observed that transplanted cells have nephrotoxic effects *per se*. This is evident in the “marrow infusion syndrome”, a dysfunction that is caused by the cryopreservants, (*i.e*., DMSO) that are commonly used for long-term storage of the hematopoietic stem cells before their transplantation. Residues of those reagents can be infused together with the stem cells, leading to hemolytic effects that eventually hamper renal functionality.

Another severe transplant complication observed in both autologous and allogeneic HSCT recipients is the transplant-associated thrombotic microangiopathy (TA-TMA), an endothelial injury syndrome, with a still unclear etiology, which leads to microangiophatic hemolytic anemia, intravascular platelet activation, and formation of thrombi within the microcirculation, with end-organ damage from ischemia, particularly in the kidneys. Among possible causes of TA-TMA, there are HSCT treatments, such as radiation, chemotherapy, calcineurin inhibitors, and some HSCT-associated complications, including viral infections, GVHD, and hepatic sinusoidal obstruction syndrome (72, 86–88). The recognition of the early stages of TA-TMA is often difficult and, if not promptly diagnosed and treated, it commonly leads to permanent renal injury and, possibly, death.

Patients undergoing transplant are at higher risk of infection due to the administration of immune suppressants. Sepsis has been ascribed as a HSCT complication that can damage renal functionality due to renal hypoperfusion, tubulointerstitial injury, and capillary thrombosis (89, 90). Patients who require treatment in intensive care unit after transplant have higher risks of developing AKI (76). Adenovirus infections and the reactivation of the BK (Polyomavirus hominis 1) virus, which can occur in HSCT recipients, can cause tubulointerstitial nephritis and cystitis (70, 91, 92).

Even prophylaxis measurements used to prevent infections can provoke renal failure, as has been reported in patients treated with antifungals, such as amphotericin B (78) and with aminoglycoside antibiotics (93).

Moreover, patients receiving allotransplant are at high risk of developing GVHD that can cause systemic detrimental effects, including nephrotic syndrome, transplant-associated microangiopathy and glomerulonephritis, most likely due to the excessive release of pro-inflammatory cytokines such as TNF-α, IL-6, and TGF-β (72, 88, 94, 95). To prevent GVHD and treat chronic GVHD, patients receive immunosuppressants, including steroids and calcineurin inhibitors, which sometimes need to be taken for prolonged periods of time, causing renal complications (71, 96, 97).

An impairment of kidney activity that is a quite common primary or secondary complication in HSCT patients is at the base of an altered systemic mineral homeostasis, and, indirectly, of bone metabolism, that could be further worsened if it is associated with malabsorptive syndromes.

### 2.2 Bone loss

Bone homeostasis is the result of a finely regulated and correctly balanced activity of two different types of cells: osteoblasts, which form new mineralized bone, and osteoclasts, which reabsorb old and/or damaged bone tissue. Alterations in this homeostasis lead to the development of two main classes of bone diseases: those characterized by an excess of new bone deposition and mineralization, and those characterized by increased bone mass loss and/or reduced mineralization. Ultimately, both these two classes of disease generate a structurally impaired bone tissue, with altered mechanical properties, prone to deformities and/or fragility fractures.

Recent studies have reported that alteration of bone homeostasis, with loss of bone mass (osteopenia, osteoporosis), is a common side effect of the HSCTs (98–100). Osteopenia is diagnosed in 50% of cases 4-6 years post HSCT, while osteoporosis is diagnosed in 20% (98). It has been reported that the loss of bone mass occurs already few months after HSCT, with patients showing a reduction in Bone Mineral Density (BMD) primarily at femoral neck and lumbar spine (101, 102). Transplanted patients have an overall higher fracture risk compared to the general population (103, 104). Pathogenesis of accelerated bone loss in HSCT recipients is a multifactorial dysfunction that is not yet completely understood due to its complexity and to the contribution of many factors that concomitantly impact bone homeostasis (**Figure 1**) (105–108). These multiple factors include the treatments that HSCT patients undergo before, during, and after transplantation (108). Underlying hematological diseases may also contribute to osteoporosis *per se* by inhibiting osteogenesis and promoting bone resorption, thus predisposing HSCT-patients to higher risks of BMD loss (109, 110). The age at transplantation appears to be a risk factor itself for bone mineral deficit in recipients of HSCT. Indeed, HSCT patients less than 10 years were showed to have, at an average age of 14 years after the transplantation, significantly lower Z-scores at lumbar spine and femur neck than HSCT patients transplanted at over 18 years and non-transplanted sibling controls (111).

Pre-transplant myeloablative treatments have been shown to negatively impact bone metabolism. Chemotherapeutic compounds, such as alkylating agents, methotrexate, and doxorubicin can deregulate bone remodeling by directly inhibiting osteoblast proliferation and osteoblastogenesis and, simultaneously, by stimulating osteoclast activity and bone resorption (112–116), or by indirectly damaging the reproductive system and sex hormone metabolism, which play a fundamental role in regulating skeletal modeling and life-long remodeling of bone tissue (117–120).

Total body irradiation, often coupled with chemotherapy as myeloablative conditioning treatment, dramatically reduces BMD by suppressing bone formation, enhancing bone resorption, and disrupting the inorganic components of the bone matrix (121–125). Moreover, irradiation has been associated with hypogonadism, hypopituitarism and growth hormone deficiency, thus leading to a decrease in trophic factors important for bone formation (105, 126, 127).

Chemotherapy- and/or radiotherapy-caused hypogonadism is one of the main causes of the occurrence of osteoporosis in HSCT patients. Indeed, under physiological conditions, sexual hormones are among the major regulators of bone metabolism in both females (estrogens and progesterone) and males (testosterone) (128).

Estrogens have a positive activity on bone formation, both by inhibiting osteoblast apoptosis and by increasing their lifespan (129). Consequently, the absence of these hormones in hypogonadal HSCT female patients promotes a bone metabolic framework oriented towards resorption. At the molecular level, estrogen absence leads to the non-activation of Src/Shc/ERK signaling and, consequently, the promotion of JNK, which, by modifying the activity of a series of transcription factors, including c-Jun/c-Fos, Elk-1, and CCAAT enhancer binding protein-β (C/EBPβ) (130, 131), promotes osteoblastic apoptosis. In addition, estrogen deficiency leads to a clear increase in NF-κB activity in osteoblastic cells (132), leading to a possible decrease in the expression of Fos-related antigen-1 (Fra-1), a transcription factor essential for bone matrix formation (131), contributing to a deficit in bone anabolism. At the same time, the estrogen deficiency leads to a fourfold increase in bone resorption activity (133, 134); the absence of estrogen causes a rapid decrease in bone mass due to the expansion of T-cells that produce high levels of TNFα in the bone microenvironment. TNFα, together with activation of macrophage colony-stimulating factor (M-CSF) and RANK, activates its p55 receptor, promoting osteoclastogenesis (135–138). Osteocytes respond to estrogen deficiency; the absence of estrogen increases osteocyte apoptosis, resulting in increased concentrations of RANKL, probably released by apoptotic osteocytes or as a result of cell-to-cell signaling between healthy and apoptotic osteocytes, which induces increased bone resorption through stimulation of the osteoclastic cell compartment (131, 139–142).

Testosterone deficiency is a known cause of bone loss and the onset of osteoporosis in men (143–153). Chronic testosterone deficiency, such as that occurring in hypogonadal HSCT male patients, could concur with the early-onset osteopenia/osteoporosis observed in these individuals.

Indeed, under physiological conditions, testosterone acts on bone metabolism in two different ways:

1) directly, in its active form, dihydrotestosterone (DTH), *via* interaction with androgenic receptors (ARs) present on osteoblasts, activating the TGF-β signaling pathway, and, thus, promoting osteoblastic proliferation and osteoblastogenesis (154, 155),
2) indirectly, after its conversion to estradiol (E2), by binding the estrogen receptor alpha (ERα), and exerting a local positive action on osteoblast function, exactly as systemic estrogens do in women.

Testosterone deficiency results in a decreased differentiation and activity of osteoblasts, leading to decreased new bone formation. Orchiectomized rodents showed that testosterone depletion induces a significant increase of RANKL and IL-6 expression in bone marrow-derived stromal cells, which strongly promote osteoclast differentiation, with consequently increased bone resorption and bone mass loss (156–158). Moreover, testosterone deficiency causes an indirect deficiency of estradiol (159, 160), which leads to an enhanced bone loss through the increased production of several inflammatory cytokines (IL-1 and TNFα, IL-6), which stimulate osteoclastic differentiation and activity and promote osteoblastic apoptosis (161–165).

Moreover, since testosterone plays a predominant role in male muscle strength and physical performance, it is conceivable that the absence of this hormone in hypogonadal HSCT male patients can cause sarcopenia, leading to greater weakness and increasing the risk of falling and fracturing (140, 166).

In relation to what has been described above regarding the importance of sex hormones in maintaining bone homeostasis, the onset of hypogonadism in HSCT patients obviously contributes to the loss of bone mass and the onset of osteoporosis; a greater relationship was found between individuals suffering from HSCT-derived medium/severe hypogonadism and decreased BMD and osteoporosis (167–169).

Several studies have reported that one early side effect of HSCT is the failure of bone morphogenesis, due to a severe reduction of osteoblast progenitor cells in bone marrow, causing post-transplant BMD loss (106, 108, 170–172). Moreover, the immune response, following the transplant, seems to play a pivotal role in altering bone homeostasis by promoting osteoclast maturation and activity (105, 106, 108). After HSCT, indeed, it has been observed that the balance between RANKL and osteoprotegerin (OPG), expressed/released by bone marrow stromal cells and osteoblasts, is shifted towards RANKL expression, causing an excessive induction of osteoclast differentiation and activity, with increased bone resorption. Simultaneously, it has been seen that certain cytokines typical of the immune and inflammatory response, such as granulocyte-macrophage colony-stimulating factor (GM-CSF), IL-6 and TNF-α can stimulate osteoclastic activity (173–175).

HSCT-derived immune derangement has been found to trigger bone loss, most frequently in allogeneic HSCT recipients, compared to autologous HSCT, due to the increased synthesis of specific inhibitors of the canonical Wnt/Beta Catenin pathway, Dickkopf-1, and Sclerostin (176), principally as a consequence of GVHD and/or administration of GVHD prophylactic treatments. It has been observed that GVHD occurs in 40-70% of allogeneic HSCT recipients and that the severity of GVHD is positively correlated with the degree of BMD reduction (106, 173, 174, 177–179). Anti-GVDH treatments, consisting in the administration of glucocorticoids and calcineurin inhibitors, also seem to play a major role in HSCT-associated osteoporosis (107, 108). High-dose glucocorticoid therapy is usually the first-line approach against GVDH, causing a rapid decrease of BMD in HSCT-recipients, by inhibiting new bone formation and promoting bone resorption (107, 180). Glucocorticoids can shift the ratio between OPG and RANKL towards the RANKL expression, with a consequent boost in bone turnover, in a time- and dose-dependent manner (102, 180–182). Similar to glucocorticoids, immune-suppressive calcineurin inhibitors, such as cyclosporin A and tacrolimus, seem to promote bone resorption and osteoclast activity, while both interfere with osteogenesis and osteoblasts activity (104, 183).

## 3 HSCT-derived endocrine sequelae management

### 3.1 Altered mineral homeostasis and renal dysfunction

#### 3.1.1 Surveillance

General recommendations for the mineral homeostasis restoration include calcium intake and vitamin D supplementation (184, 185), together with a balanced diet, no smoking, alcohol taken in a controlled manner (≤2 alcohol units per day) and regular exercise. Recommended doses of calcium range from 1000 to 1200 mg/day of elemental calcium; if the dietary intake is insufficient, supplementation with calcium citrate or calcium carbonate is recommended.

Vitamin D deficiency can be treated with 50,000 IU of vitamin D3 once a week (or 7,000 IU/day) for 8-12 weeks to achieve serum vitamin D levels >20 ng/mL, followed by maintenance by intake of ≥1000 IU/day (186, 187). Management of CKD is based on slowing disease progression and minimizing possible further renal damage.

Screening by the primary care physician is essential for early diagnosis because he is aware of the patient’s comorbidities and has opportunities to assess changes in GFR levels. Hypertension, use of nephrotoxic agents, presence of cardiovascular disease, hyperglycemia, and increased albuminuria (188), along with age, obesity, smoking, and sex, are indices of increased risk factor for CKD. There are guidelines such as the KDIGO (189) or JNC8 (190) that provide information for screening for the disease.

Hypertension is important to keep under control in patients with C KD. Guidelines recommend keeping blood pressure below 140/90 mm Hg (190, 191), or 130/80mm Hg in the presence of albuminuria (if albuminuria levels exceed 300 mg/24, an angiotensin receptor blocker should also be taken) (191). Hemoglobin A1c should be around 7% to prevent hyperglycemia from infecting the kidney (191), sodium levels should not exceed 2000mg/d, and protein intake should be less than 0.8/kg/d (191).

Anemia can be a common complication of CKD. When GFR decreases, erythropoietin is most likely to decrease at the same time, since 80% of that protein, comes from renal interstitial cells, so with GFR values <60 and hemoglobin<10g/dL, subcutaneous erythropoietin treatment is administered (188).

Identification of risk factors, early diagnosis, and treatment of renal dysfunction are crucial for favorable outcomes. In addition, detection of subclinical renal dysfunction before transplantation can help in the choice of conditioning type so that it is less nephrotoxic.

#### 3.1.2 Treatment

The study of individualized therapy, based on the calculation of body measurements, plasma-level drug metabolite concordances, and analysis of the patient’s renal function can lead to the decreased toxicity of conditioning regimens.

Cure of CKD after HSCT is very difficult to achieve, but if a specific cause is identified, it can be cured. The use of pamidronate, for example, can cause focal glomerulosclerosis; discontinuation of the drug can promote proteinuria decrease and halt disease progression (188).

The American Society for Blood and Marrow Transplantation has published recommendations on dose modification of chemotherapeutics used in conditioning regimens that are nephrotoxic or eliminated by the kidneys (79). Fludarabine, for instance, a drug commonly used in non-myeloablative regimens for patients with comorbidities, including renal failure, is not nephrotoxic, but being eliminated by the kidneys, it may lead to an increased risk of neurotoxic adverse effects in case of renal failure (72). Excessive plasma concentrations combined with low clearance of F-Ara-A, the active metabolite of fludarabine, have been shown to be linked to increased mortality without relapse in transplanted patients, while its high clearance leads to an increased susceptibility for GVHD (192). Consequently, a dosage reduction of 20-25% is recommended (72). Clofarabine, on the other hand, should be abolished for patients over 60 years with creatinine clearance <60 mL/min, while a dose reduction of 50% is recommended in patients with CrCl between 30 and 60mL/min (79). Prolonged mucositis has been observed with standard doses of melphalan in patients with CKD, therefore, the recommended dosage would be between 100 and 140 mg/m2 for dialysis-dependent patients with renal impairment (79). The bisulfan dosage is unmodified, in fact, its renal excretion is slight even for patients in dialysis (79).

Research is focusing on the study of TA-TMA pathology and renal GVHD through the study of specific non-invasive markers. Markers of endothelial damage such as thrombomodulin, plasminogen activator inhibitor type 1 and endothelial microparticles, and inflammatory cytokines IL-6, IL-15 and monocytin.

Other areas of research include understanding the pathology of TA-TMA and hypothesized renal GvHD and finding specific noninvasive markers and therapeutic options for both complications. Particles that are hypothesized to be important are markers of endothelial damage, such as thrombomodulin, plasminogen activator inhibitor type 1 and endothelial microparticles, as well as inflammatory cytokines: IL-6, IL-15, monocyte cytokine and monocyte chemoattractant protein-1 (193).

A prognostic marker for cutaneous GVHD, elafin, when present at the urinary level, has also been found to be a prognosis maker for AKI and CKD (193). It has been investigated that the decrease in renal vascular endothelial growth factor concentration, correlated with the subsequent serum elevation of neutrophil extracellular traps, may be related to TA-TMA risk (194), consequently, supplementation of this growth factor could be valid as a therapeutic strategy.

### 3.2 Bone loss

#### 3.2.1 Surveillance

In 2006, guidelines for bone health screening and prevention of bone loss in HSCT survivors were published by the EBMT, the Centre for International Blood and Marrow Transplantation Research (CIBMTR), and the American Society for Blood and Marrow Transplantation (ASBMT) (108, 195), and successively revised and updated in 2012 (108, 196).

**Figure 2:**
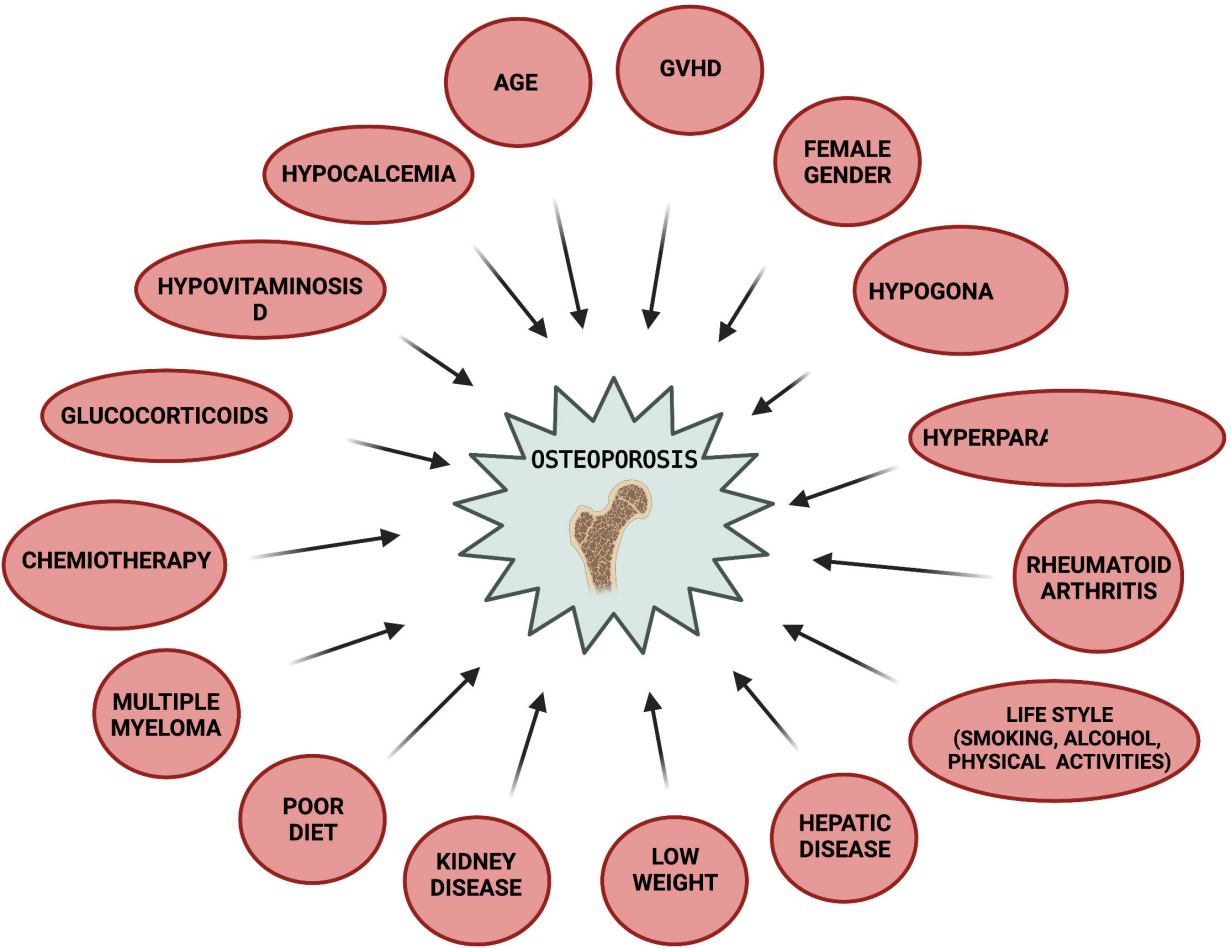
Risk factors for post-HSCT osteoporosis. Created by BioRender.com.

The general assessment of bone health status in patients suitable for HSTC should include a pre- transplant evaluation of: 1) patient and familial medical history, 2) physical examination, 3) evaluation of common modifiable osteoporosis risk factors to which the patient is exposed (197), 4) FRAX fracture risk index (184), 5) DXA at hip and lumbar spine (especially in adult women, and patients undergoing a long-term therapy with corticosteroids. In the latter ones a DXA evaluation should be planned by 3 months after the beginning of pharmacological treatment), 6) spine X-rays or vertebral fracture assessment by DXA (VFA) in older patients and high-risk patients, to identify vertebral fractures Risk factors for osteoporosis to be evaluated in HSCT patients include: age, female gender, smoking, alcohol, no physical activity, low weight, poor diet, hypogonadism, hyperparathyroidism, rheumatoid arthritis, liver and/or kidney disease, multiple myeloma, chemotherapy, glucocorticoid intake (dose- and time-dependent), and GVHD (**Figure 2**) (105, 196, 198–202). Post-transplant follow-up by DXA is recommended as early as 3 months after HSCT, and then annually, in patients at high risk (199), included those in treatment con corticosteroids, those having a pre-transplant low BMD, and/or those having experienced fragility fracture before HSCT (184, 203, 204). Lower risk patients may undergo DXA screening at one year after HSCT, and then on annual basis. DXA screening intervals may be reduced or extended based on post-transplant assessed BMD values, and according to the presence or absence of unmodifiable risk factors (older age, female gender, previous fragility fracture) and modifiable current risk factors.

#### 3.2.2 Treatment

Given the heterogeneity of HSCT patients in age, gender, and bone response to transplant and associated medical therapies, there are not standard treatment guidelines for bone in these patients (108, 205). Current therapeutic strategy for osteoporosis is, thus, the one indicated for all organ-transplanted patients (108). In general, the first 3 months after transplantation are the most at risk of complications and, consequently, any drug therapy not directly aimed at the success of the transplant is precluded to avoid further interactions and side effects, including anti-fracture drugs. After 3 months, if the patient’s medical conditions permit, and after a monitoring of renal and hepatic status, the guidelines allow treatment for the prevention/cure of bone loss (108, 205).

There are two types of approaches proposed for the prevention/treatment of bone loss. The first is called “treat to target” (205) and consists of treating patients with a therapy according to the target to be achieved; if this is not achieved, the therapeutic approach is changed until the most appropriate therapy is found for the patient (206). The second approach is usually applied to patients who cannot undergo the first approach (t-score <-3; medical complications; contraindications for a type of drug, etc.), and consists of a bisphosphonate oral therapy for 5 years, or intravenous administration for 3 years, followed by a discontinuation of treatment for at least 2 years, to be resumed if necessary (207). Oral treatment is generally the first to be prescribed, unless there is intestinal GVHD, esophageal dysmotility, or gastrointestinal reflux disease. Alternatively, intravenous zoledronate administration may be indicated, but must be strictly avoided in hypocalcemic patients or those with CDK stage 3B-5 (205). Bisphosphonate therapy has been shown to be safe for HSCT survivors (208–211), in improving BMD (208–211). One rare side effect of bisphosphonates, principally for Zoledronate (1% of treated cases), is the osteonecrosis of the jaw (212, 213). Since HSCT survivors with chronic oral GVHD are at increased risk (214), a complete oral examination and the complete elimination of oral infections, which represent major risk cofactors for this pathological manifestation, are strongly recommended prior to therapy with bisphosphonate (213, 215, 216).

Estrogen replacement therapy is considered the best treatment for bone loss associated with early menopause (217), like that experienced in hypogonadal HSCT female patients; this therapy should be limited only to subjects not at risk for breast cancer (218). Transdermal administration is preferred as it carries a lower risk of thromboembolism and stroke (219). Once the age of natural menopause has been reached, discontinuation of therapy is customized, as it has been shown that the benefits for the patient outweigh the risks in the first 10 years after menopause (220–223).

In hypogonadal HSCT male patients presenting low testosterone levels, testosterone replacement therapy is highly recommended, and it can also reduce the risk of early-onset osteoporosis occurrence (108, 144, 205, 224, 225).

Calcitonin nasal spray therapy reduces the risk of fractures but is not used as a routine drug because it is less effective than bisphosphonates (226, 227); it is only prescribed in patients who cannot receive bisphosphonates, or for patients with acute and painful fractures, because of the analgesic effect of calcitonin (227).

The beneficial effect of denosumab in HSCT patients is not yet well understood. There is one study in which 33 HSCT female patients with osteoporosis, who were naïve for anti-fracture drugs, were treated with denosumab (60 mg) three times every six months (0, 6, and 12 months). The BMD of the lumbar spine, femoral neck and total hip, measured 12 months after the first administration of denosumab, showed an improvement of 4.39 ± 6.63%, 3.11 ± 7.69% and 1.97 ± 6.01%, respectively, with respect to baseline, in the absence of serious adverse reactions to the point of discontinuing therapy. Data from this study suggested that denosumab could be an efficient therapy for the prevention/treatment of bone loss in HSCT recipients (228).

Currently, there are no conclusive data on safety and efficacy of teriparatide, raloxifene, or romosozumab for bone loss management in HSCT recipients.

Calcium and vitamin D supplementations are usually prescribed in combination with drug treatment (186, 187).

Medical therapy for bone loss in children surviving HSCT is similar to that performed for adults, but more oriented towards estrogen and growth hormone replacement therapy (229–233). Treatment with bisphosphonates is performed following the “treat to target” approach and is reserved for children with osteoporosis who do not respond to calcium and vitamin D supplementations, and estrogen and/or growth hormone replacement therapies (205). Teriparatide is not used, while calcitonin is administered as short-term adjunctive therapy (234).

In our clinical experience, we use the two most common anti-resorption drugs, either bisphosphonates or denosumab, for the prevention/treatment of early and/or increased bone loss in HSCT patients.

## 4 Discussion

Thanks to improvements in patient care and the implementation of more tolerable conditioning treatments and effective prophylaxis, long-term survival of HSCT has increased significantly in recent years. However, concomitant with the increase in survival after HSCT, complications, related to the transplant itself and/or to pre- and post-transplant required medical treatments, are emerging, and numerous retrospective and follow-up studies have highlighted the occurrence of multiple side effects that worsen patient quality of life and increase the risk of severe morbidity and mortality. Among these side effects, disorders affecting the endocrine systems are common after HSCT, including metabolic, growth, and sexual defects, and mineral metabolism alterations.

Among side effects of HSCT, the malfunctioning of calcium-tropic organs, such as kidneys, bones, and intestines, has proven implications for systemic mineral homeostasis, which can in turn affect the entire skeletal metabolism by causing increased bone resorption, leading to the onset of early osteoporosis and bones prone to fragility fracture in a wide percentage of HSCT survivors. Bone health management needs to be refined to better tailor osteoporosis therapies, specifically for HSCT recipients. The recent arrival in the market of romosozumab antibody will be an important addition in the pharmacological armamentarium of the physician taking care of bone fragility in these patients.

An interesting aspect that has been observed in few studies is that the alteration of the systemic mineral homeostasis following HSCT, also powered by post-transplant chronic glucocorticoid intake, affects the entire phosphate-calcium-PTH system by altering PTH secretion and causing the onset of secondary hyperparathyroidism, which, if not rebalanced, can lead to the development of chronic tertiary hyperparathyroidism. The presence of secondary or tertiary hyperparathyroidism further contributes to worsening both kidney defects and bone mass loss and fragility. Targeted studies evaluating a possible direct effect of HSCT on parathyroid glands/cells, and the occurrence of primary hyperparathyroidism, are currently lacking.

## Author contributions

All authors listed have made a substantial, direct, and intellectual contribution to the work and approved it for publication.
